# Effects of Glucose Concentration on Propofol Cardioprotection against Myocardial Ischemia Reperfusion Injury in Isolated Rat Hearts

**DOI:** 10.1155/2015/592028

**Published:** 2015-09-28

**Authors:** Xinhua Yao, Yalan Li, Mingzhe Tao, Shuang Wang, Liangqing Zhang, Jiefu Lin, Zhengyuan Xia, Hui-min Liu

**Affiliations:** ^1^Department of Anesthesiology, Guangzhou Hospital of Traditional Chinese Medicine, Guangzhou 510130, China; ^2^Department of Anesthesiology, The First Affiliated Hospital of Jinan University, Guangzhou 510630, China; ^3^Department of Anesthesiology, Affiliated Hospital of Guangdong Medical College, Zhanjiang, Guangdong 524001, China; ^4^Department of Anesthesiology, The University of Hong Kong, Hong Kong; ^5^Department of Anesthesiology, Renmin Hospital of Wuhan University, Wuhan 430060, China

## Abstract

The anesthetic propofol confers cardioprotection against myocardial ischemia-reperfusion injury (IRI) by reducing reactive oxygen species (ROS). However, its cardioprotection on patients is inconsistent. Similarly, the beneficial effect of tight glycemic control during cardiac surgery in patients has recently been questioned. We postulated that low glucose (LG) may promote ROS formation through enhancing fatty acid (FA) oxidation and unmask propofol cardioprotection during IRI. Rat hearts were isolated and randomly assigned to be perfused with Krebs-Henseleit solution with glucose at 5.5 mM (LG) or 8 mM (G) in the absence or presence of propofol (5 *μ*g/mL) or propofol plus trimetazidine (TMZ). Hearts were subjected to 35 minutes of ischemia followed by 60 minutes of reperfusion. Myocardial infarct size (IS) and cardiac CK-MB were significantly higher in LG than in G group (*P* < 0.05), associated with reduced left ventricular developed pressure and increases in postischemic cardiac contracture. Cardiac 15-F2t-isoprostane was higher, accompanied with higher cardiac lipid transporter CD36 protein expression in LG. Propofol reduced IS, improved cardiac function, and reduced CD36 in G but not in LG. TMZ facilitated propofol cardioprotection in LG. Therefore, isolated heart with low glucose lost sensitivity to propofol treatment through enhancing FA oxidation and TMZ supplementation restored the sensitivity to propofol.

## 1. Introduction

Ischemic heart disease is a major cause of mortality, and the global incidence is rising rapidly. Essentially it is a reduction of heart blood flow usually secondary to atherosclerosis which then reduces the delivery of oxygen and nutrients. Rupture of vulnerable arterial plaques and subsequent thrombosis results in myocardial infarction (MI) and this is one of the commonest perioperative complications after noncardiac surgery [1], especially in patients with diabetes [2–4]. In nonoperative settings, reperfusion therapies currently remain the mainstay therapy for the ischemia results from coronary occlusion. Reperfusion strategies, such as thrombolysis, angioplasty, and percutaneous coronary intervention (PCI), when applied expeditiously, and coronary artery bypass grafting (CABG) can restore coronary flow and limit myocardial infarct size (IS), and reperfusion per se can paradoxically trigger irreversible tissue injury, namely, ischemia-reperfusion injury (IRI) which is regarded as an important contributor to the unsuccessful recovery after reperfusion therapy [5, 6]. The increased reactive oxygen species (ROS) production during reperfusion and the subsequently increased oxidative damage are regarded as a pivotal factor contributing to the pathogenesis of IRI [7, 8].

To date, a large body of the literature supports the concept that disturbances in cardiac energy metabolism have been involved in exaggerating IRI, in contrast to normal heart where fatty acid and glucose metabolism, specifically fatty acid (FA) oxidation and glucose oxidation, are tightly regulated [9]. Administering a high level of FA in healthy hearts can inhibit glucose oxidation and exacerbate IRI [10]. During ischemia, glucose becomes the preferred cardiac energy substrate, as it requires less oxygen for oxidation than an equimolar amount of carbon derived from FA. The metabolic shift between FA oxidation and glucose oxidation has been proposed to be the key contributor in the pathogenesis of impaired cardiac function in ischemic reperfused hearts [11] and a recent study further demonstrates that the fatty acid transporter, FAT/CD36, plays critical roles in coordinating changes in cardiac substrate metabolism during ischemia and reperfusion [12]. Clinical data shows that patients with diabetes are more frequently encountered in ischemic heart disease and more vulnerable to IRI than nondiabetics and hyperglycemia-induced oxidative stress may further exacerbate IRI [13]. In addition, cardiac energy metabolism in diabetic hearts is altered where glucose utilization is impaired which causes a switch to predominantly using FA for energy supply [14]. This metabolic maladaptation, resulting from elevated plasma free fatty acids, excessive FA accumulation, and increment of mitochondrial uncoupling and free radical accumulation, will provide feedback to inhibit glucose uptake, which is detrimental to the ischemic myocardium and amplifies deleterious effects [10, 15] and might be the fundamental mechanism governing the increased vulnerability of the diabetic heart to IRI. In line with this mechanism, switching the energy supply preference away from FA oxidation and subsequently to greater glucose oxidation has been reported to be cardioprotective in lessening myocardial IRI [16].

Accumulating evidence supports that administration of insulin to maintain blood glucose at a level below 215 mg/dL can improve long-term survival in diabetic patients suffering from acute myocardial infarction [17–19]. Consistent with this finding, tight glycemic control with glucose-insulin-potassium (GIK) solution that normalized serum glucose concentration from 125 to 200 mg/dL has been shown to significantly decrease the morbidity in diabetic patients undergoing CABG surgery [16]. Further lowing blood glucose values to no higher than 110 mg/dL with intensive insulin therapy has also been found to be beneficial among critically ill patients [20]. However, the beneficial effects of tight glycemic control during cardiac surgery in patients remain questioned and controversial as conflicting reports have emerged. In contrast to aforementioned studies, other researches demonstrated that intensive blood glucose control has no effect on reducing mortality in patients with type 1 or type 2 diabetes [21] and significantly increased the risk of intraoperative hypoglycaemia without clear postoperative benefit in patients undergoing cardiac surgery using cardiopulmonary [22]. So far, to our knowledge, there is no direct evidence to explore the effects and mechanism of tight glycemic control during myocardial IRI.

Propofol, a commonly used intravenous anesthetic, has antioxidant property, which enhances the capacity of endogenous cardiac antioxidant to inhibit ROS-mediated lipid peroxidation [23, 24] and attenuates postischemic myocardial cellular injury and oxidative stress in patients undergoing cardiac surgery [25] and in rat hearts subjected to global myocardial IRI [26]. However, controversy about propofol cardioprotection persists. For example, propofol failed to attenuate ROS production during ischemia reperfusion in the isolated rat heart [27]. The precise reasons for different outcomes are as yet unknown. Furthermore, a study shows that propofol cardioprotection was abrogated in diabetic condition [28]. However, study regarding the effectiveness of propofol at different glucose concentrations in combating myocardial IRI has not been elucidated. In particular, studies addressing whether or not the cardioprotective effect of propofol is related to FA oxidation and lipid peroxidation production are lacking. To examine this, we use low glucose concentration at 5.5 mM (which is equal to 100 mg/dL) to mimic tight glycemic control condition [20] and 8 mM as normal glucose (which is about the normal high postmeal glucose level) in isolated ischemic perfused rat heart model to explore the impact of glucose concentration on propofol cardioprotection against myocardial IRI, and we incorporated the use of trimetazidine (TMZ), a partial FA oxidation inhibitor to explore the effect and mechanism of inhibiting FA metabolism in attenuating postischemic oxidative stress and myocardial IRI at varying glucose concentrations and its potential impact on propofol cardioprotection.

## 2. Materials and Methods

### 2.1. Experimental Protocol

Hearts were randomly assigned to one of the six experimental groups (*n* = 7 per group): low glucose concentration at 5.5 mM (LG), LG plus propofol (5 *μ*g/mL) (LG+P), LG plus propofol together with the partial FA oxidation inhibitor trimetazidine (TMZ) (1 *μ*M) (LG+P+TMZ), glucose concentration at 8 mM (G), G plus propofol (G+P), and G plus propofol together with TMZ (G+P+TMZ). The propofol concentration of 5 *μ*g/mL was chosen based on our previous finding that propofol used at this concentration can confer cardioprotection in the isolated ischemic reperfused rat hearts [26]. After 20 minutes of stabilization (baseline, BS20), the heart was subjected to 35 min global ischemia by turning off the perfusion and followed by 60 min reperfusion using Langendorff-perfused system. The study drugs propofol and TMZ were perfused through the aorta via a minipump. The LV pressure signal was recorded using a PowerLab data acquisition system and processed to yield left ventricle developed pressure (LVDP) calculated from left ventricular end-diastolic pressure (LVEDP) and LV peak systolic pressure (LVSP) (LVDP = LVSP − LVEDP). Coronary perfusion pressure (CPP) was measured via a side arm of the perfusion cannula connected to a pressure transducer as described [29]. Effluent samples during baseline, ischemia, and reperfusion were collected at different time points for analysis for creatine kinase MB (CK-MB), endothelin-1 (ET-1), and free 15-F2t-isoprostane (15-F2t-IsoP) concentration described as below. Heart tissue protein expression of CD36, a cardiac lipid transporter, was measured by Western blotting.

### 2.2. Global Ischemia by Langendorff-Perfused System in* Ex Vivo*

Male Sprague-Dawley rats (250 ± 10 g) were used at approximately 6 weeks of age from the Laboratory Animal Service Centres, the University of Hong Kong and Wuhan University, China. Rats were housed and given free access to standard food and water according to the principles of Animal Care of the University of Hong Kong. Experiments were approved by the Committee on Use of Live Animals in Teaching and Research (CULATR). Rats were anaesthetized by injection of intraperitoneal pentobarbital sodium (65 mg/kg). Median thoracotomy was performed and hearts were quickly excised and immersed in ice-cold Krebs-Henseleit (KH) solution. Hearts were gently squeezed to remove residual blood to prevent clot formation. Then, hearts were cannulated via the aorta and perfused as nonworking “Langendorff” method. The perfusate at a constant flow rate of 10 mL/min which was KH solution that contained (in mM) NaHCO_3_ 24, NaCl 118, MgCl_2_ 1.2, CaCl_2_ 1.25, KCl 4.63, KH_2_ PO_4_ 1.17, and glucose 5.5 or 8 was previously equilibrated with a mixture of 95% O_2_^−^ and 5% CO_2_ at 37°C using a thermostatically controlled water circulating system. The heart was kept warm in a chamber with circulating water controlled at 37°C. A water-filled balloon was then inserted into the left ventricle through the mitral valve and connected to a pressure transducer for the determination of LV pressure. The balloon was inflated to a diastolic pressure of ~5–10 mmHg.

### 2.3. Myocardial Infarct Size (IS)

At the end of reperfusion, 1% 2,3,5-triphenyl-tetrazolium chloride (TTC) reaction (Sigma Chemical Co., St. Louis, MO) in phosphate buffer at pH 7.4 was pumped into the heart until the epicardial surface became deep red. Then tissues were fixed overnight in 10% formalin. The infarcted areas (white) and viable areas (red) for each slice were traced and digitized using a computerized planimetry technique (SigmaScan 4.0, Systat Software Inc., Richmond, CA). The ratio of infarct size (white)/total area (white plus red) was used to compare the differences among groups.

### 2.4. Creatine Kinase MB (CK-MB)

Cytoplasmic enzyme CK-MB reversibly catalyzes the reaction from ATP and creatine to phosphocreatine and ADP. Increased CK-MB concentration is used as an index of myocardial cell injury. The level of CK-MB was determined using a commercial ELISA assay kit (R&D Systems, Minneapolis, MN) according to the manufacturer's instructions as described [30]. Briefly, effluent perfusate was collected at 20 min after baseline stabilization (BS20) and at 1 (Re-1) and 60 (Re-60) min of reperfusion and assayed in a 96-well plate precoated with antibody specific to CK-MB. The color was measured by the absorbance under the excitation of light at 450 nm. The concentration of CK-MB was determined by comparing the absorbance of each sample to the standard curve and expressed as ng/mL.

### 2.5. Endothelin-1 (ET-1)

Measurement of ET-1 concentration in the coronary effluent sampled at BS20 and 1 (Re-1) and 60 (Re-60) min of reperfusion was performed using a commercially human ET-1 EIA kit 900-020 (Assay Designs, Inc., Ann Arbor, MI) as described [31]. After collected effluent samples were initially concentrated 4-fold by evaporation of KH solution under a stream of dry nitrogen, they were assayed in 96-well microplates according to enzyme immunoassays for analysis of ET-1 concentration. Thereafter, the plates were read at 450 nm and the concentration of ET-1 was expressed as picograms ET-1 per milliliter effluent.

### 2.6. Free 15-F2t-Isoprostane (15-F2t-IsoP)

15-F2t-isoprostane (15-F2t-IsoP), a specific marker of oxidative stress and lipid peroxidation, is produced by oxidation of tissue phospholipids and can be detected by using a commercial available competitive enzyme immunoassay kit as described [32]. The effluent samples collected at BS20 and 1 (Re-1) and 60 (Re-60) min of reperfusion were assayed relying on the competition between free 15-F2t-IsoP and 15-F2t-IsoP-acetylcholinesterase conjugate (15-F2t-IsoP tracer) for a limited number of 15-F2t-IsoP specific rabbit antiserum binding sites. The rabbit antiserum free 15-F2t-IsoP complex can bind to the rabbit IgG mouse monoclonal antibody which was previously attached to the well to develop an optimal yellow color. The absorbance was measured spectrophotometrically at a wavelength of 412 nm. The values of 15-F2t-IsoP were expressed as pg/mL, respectively.

### 2.7. Protein Extraction and Western Immunoblotting

At the end of reperfusion, LV tissues were removed and frozen for Western blotting. The frozen LV tissues were lysed with lysis buffer and centrifuged at 13,000 g for 30 min. The supernatant was collected as isolated total protein. The determination of protein concentration was assayed as described [29]. Equal amounts of protein were separated by 10–12% SDS PAGE and transferred to polyvinylidene difluoride membranes for 1 hour. The membranes were blocked with 5% skimmed milk for 2 hours and probed with primary antibodies for CD36 and GAPDH in trisbuffered saline containing 5% BSA followed by incubation with anti-rabbit IgG conjugated to horseradish peroxidase. Specific antigen-antibody complex was detected by a standard enhanced chemiluminescence detection system (GE Healthcare, Amersham, UK). The band densities were quantified using Quantity One analysis software.

### 2.8. Statistical Analysis

Seven rats were needed in each group to detect significant differences (*P* < 0.05) in animal studies according to our previous and preliminary research. Cardiac variables and chemical assay parameters were compared by 2-way ANOVA with repeated measures (GraphPad Prism, USA). One-way ANOVA was used to test for differences in infarct size between groups. Values are presented as mean ± SEM. *P* value less than 0.05 was considered statistically significant different.

## 3. Results

### 3.1. Myocardial Infarct Size (IS) and CK-MB Release during Ischemia Reperfusion

**Figure 1:**
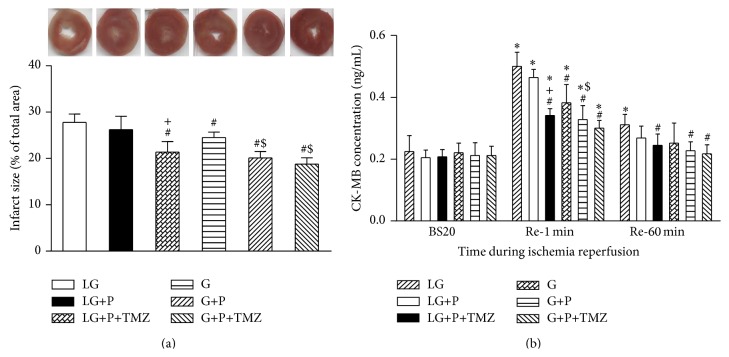
Effect of low glucose (LG) and glucose (G) on myocardial infarct size (IS) and CK-MB release during ischemia reperfusion. Rat hearts were randomly assigned to be perfused with glucose concentration at LG, LG+propofol (LG+P), and LG+propofol+trimetazidine (LG+P+TMZ) or with G, G+propofol (G+P), and G+propofol+trimetazidine (G+P+TMZ). Myocardial IS was measured by TTC staining. Myocardial IS and cardiac CK-MB release were significantly higher in LG than in G (*P* < 0.05). Propofol significantly reduced IS and CK-MB in G but not in LG. TMZ facilitated propofol induced IS and CK-MB reduction in LG but did not induce further propofol beneficial effects on G. BS20, Re-1, and Re-60 are abbreviations of 20 min after stabilization, 1 and 60 min after reperfusion, respectively. Values are presented as mean ± SEM. *N* = 7 per group. ^*^ *P* < 0.05 versus BS20; ^#^ *P* < 0.05 versus LG; ^+^ *P* < 0.05 versus LG+P; ^$^ *P* < 0.05 versus G.

Infarct size (IS) as an index of myocardial injury was expressed as a percentage of total area. The postischemic IS was significantly reduced from 28 ± 5% in the LG group to 22 ± 4% in the G group, 18 ± 5% in the G+P group, and 16 ± 3% in the G+P+TMZ group (all *P* < 0.05 versus LG, Figure 1(a)). Propofol treatment had no significant impact upon IS in the LG+P group. However, TMZ used in the LG+P+TMZ group restored the infarct sparing effect of propofol (*P* < 0.05 versus LG and LG+P, Figure 1(a)).

As shown in Figure 1(b), effluent CK-MB release did not significantly change during baseline. CK-MB release at Re-1 was significantly higher than its baseline corresponding value (all *P* < 0.05). However, at the end of 60 min reperfusion, CK-MB level was greater than its baseline value in the LG group but did not significantly differ from baseline values in other groups. Effluent CK-MB concentration in the LG+P group decreased relative to the value in the LG group at Re-1, but the decrease did not reach statistical significance (*P* > 0.05). CK-MB concentrations in all normal glucose (G) groups decreased more rapidly and were significantly lower than that in the LG group (*P* < 0.05 versus LG). The FA oxidation inhibitor TMZ preserved the effect of propofol in reducing postischemic CK-MB release in the LG+P+TMZ group (*P* < 0.05 versus LG and LG+P).

### 3.2. Contracture Development during Ischemia Reperfusion and Functional Response to Ischemia Reperfusion

**Figure 2:**
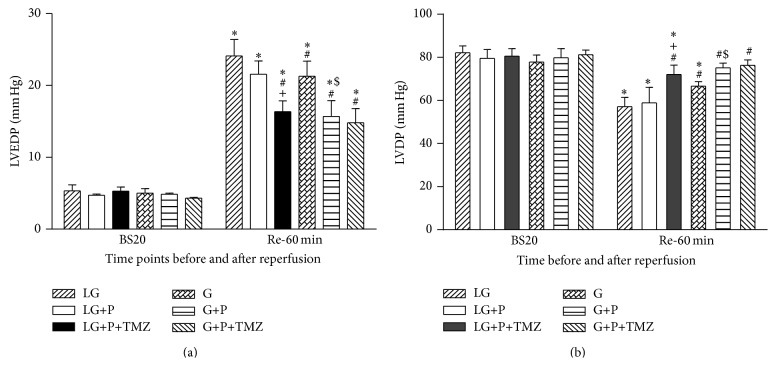
Effect of low glucose (LG) and glucose (G) on left ventricular end-diastolic pressure (LVEDP) and left ventricle developed pressure (LVDP) during ischemia reperfusion. LVEDP is not only a marker of diastolic function but also a good predictor of cardiac mortality independent of LV ejection fraction. LVDP was calculated by subtracing LVEDP, from LV peak systolic pressure (LVSP). There was significantly increase in LVEDP and decrease in LVDP in LG as compared to G (all *P* < 0.05). Propofol significantly improved cardiac function in G but not in LG. TMZ facilitated propofol cardioprotection in LG. Values are presented as mean ± SEM. *N* = 7 per group. ^*^ *P* < 0.05 versus BS20; ^#^ *P* < 0.05 versus LG; ^+^ *P* < 0.05 versus LG+P; ^$^ *P* < 0.05 versus G.

As shown in Figure 2(a), at the end of 60 min reperfusion, the magnitude of LVEDP was significantly higher than that at BS20 in all groups (*P* < 0.05). The magnitude of LVEDP in the G, G+P, and G+P+TMZ group was significantly lower than that in the LG group at Re-60. However, LVEDP value in the LG+P group did not significantly differ from that in the LG group indicating that low glucose attenuated the ischemic contracture reducing effect of propofol. TMZ treatment in the LG+P+TMZ group augmented the effect of propofol in reducing LVEDP value and led to a significant change compared to the LG and LG+P group. TMZ treatment alone tended to decrease LVEDP but did not reach significance on this change in the G+P+TMZ group.

The LVDP did not change significantly over time during baseline period and did not differ among groups. At Re-60, the LVDP values were lower than that at BS20 in both the LG and G groups as well as in LG+P groups (all *P* < 0.05). At Re-60, LVDP in the G group was significantly higher than that in the LG group (*P* < 0.05), and propofol significantly further increased LVDP in the G+P group (*P* < 0.05 versus G or LG, Figure 2(b)). In addition, propofol tended to elevate LVDP in the LG+P group, but the increase was not remarkable (*P* > 0.05 versus LG). LVDP function at Re-60 was significantly enhanced by TMZ treatment in the G+P+TMZ group which reached a level comparable to that at BS20.

### 3.3. Endothelin-1 (ET-1) Release and Coronary Perfusion Pressure (CPP)

**Figure 3:**
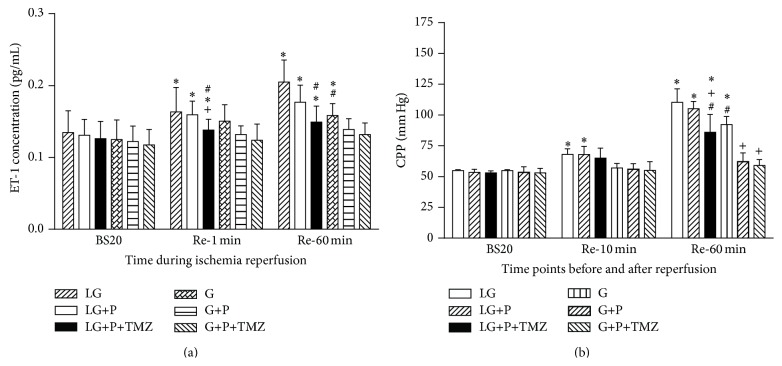
Effect of low glucose (LG) and glucose (G) on endothelin-1 (ET-1) concentration and coronary perfusion pressure (CPP). ET-1 release and CPP were significantly increased in LG as compared to G (*P* < 0.05). Propofol significantly reduced cardiac ET-1 and CPP in G but not in LG. TMZ facilitated propofol induced reduction in ET-1 and CPP in LG but did not in G. Re-10 is abbreviation of 10 min after reperfusion. Values are presented as mean ± SEM. *N* = 7 per group. ^*^ *P* < 0.05 versus BS20; ^#^ *P* < 0.05 versus LG; ^+^ *P* < 0.05 versus LG+P.

Baseline effluent ET-1 concentrations had no difference among groups. During reperfusion, ET-1 increased significantly in all LG groups at RE-1 and Re-60 as compared to baseline (*P* < 0.05 versus BS20, Figure 3(a)). Significant increase in ET-1 did not occur in the normal glucose (G) group until Re-60 and this increase in postischemic ET-1 was prevented by propofol and the combination of propofol and TMZ. Propofol plus TMZ but not propofol alone significantly attenuated the increase in ET-1 during reperfusion at RE-1 and Re-60.

As shown in Figure 3(b), during reperfusion, CPP significantly increased upon reperfusion at Re-10 and at Re-60 in the LG group and these significant increases in CPP were attenuated by propofol plus TMZ but not propofol alone. Neither propofol nor TMZ affected CPP at BS20. Significant increase in CPP was not seen in the normal glucose group until Re-60 (*P* < 0.05 versus BS20) and either propofol alone or its combination with TMZ prevented the increase in postischemic CPP.

### 3.4. 15-F2t-IsoP Generation and Cardiac CD36 Protein Expression during Ischemia Reperfusion

**Figure 4:**
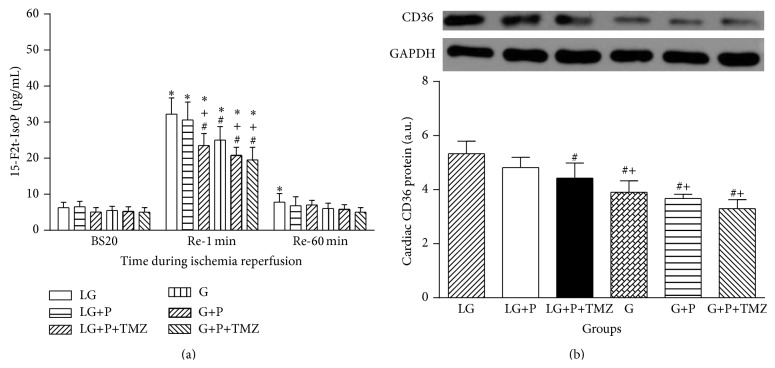
Effect of low glucose (LG) and glucose (G) on cardiac 15-F2t-isoprostane (15-F2t-IsoP) and cardiac CD36 protein expression. Cardiac 15-F2t-IsoP, a specific index of ROS-induced oxidative stress, was higher in LG that was associated with higher cardiac lipid transporter CD36 protein expression than in G. Propofol significantly reduced 15-F2t-IsoP as well as CD36 in G but not in LG. TMZ did not change the protein level of CD36 in LG. BS20 is abbreviation of 20 min after stabilization. Values are presented as mean ± SEM. *N* = 7 per group. ^*^ *P* < 0.05 versus BS20; ^#^ *P* < 0.05 versus LG; ^+^ *P* < 0.05 versus LG+P.

As shown in Figure 4, effluent 15-F2t-IsoP release had no significant change among each group at BS20. Free 15-F2t-IsoP content was dramatically increased at Re-1 as compared to that at BS20 in all groups (*P* < 0.05). Postischemic 15-F2t-IsoP levels in the G group were lower than that in the LG group at Re-1 (*P* < 0.05). Propofol treatment did not have significant effect on 15-F2t-IsoP content in the LG group (*P* > 0.1, LG+P versus LG). Propofol in combination with TMZ remarkably decreased the level of 15-F2t-IsoP in the LG+P+TMZ group when compared to the LG+P group (*P* < 0.05). Propofol treatment alone significantly reduced postischemic 15-F2t-IsoP at Re-1 in the G group (*P* < 0.05, G+P versus G), while propofol in combination with TMZ did not further decrease the level of 15-F2t-IsoP (*P* > 0.1, G+P+TMZ versus G+P).

As shown in Figure 4(b), during reperfusion at Re-60, cardiac CD36 protein expression was higher in the LG group than in the G group; propofol plus TMZ but not propofol alone reduced postischemic cardiac CD36 protein expression in the LG group (*P* > 0.05, LG+P versus LG; *P* < 0.05, LG+P+TMZ versus LG). In contrast, myocardial protein expression of CD36 was downregulated in all glucose (G) groups (all *P* < 0.05 versus LG). Propofol treatment moderately but not significantly decrease CD36 protein in the G+P group. TMZ did not further suppress the expression of CD36 in the G+P+TMZ group.

## 4. Discussion

We have shown in this study that postischemic myocardial IS and cardiac CK-MB release were significantly greater in low glucose group than in glucose group (*P* < 0.05), which was associated with reduced LVDP and an increase in postischemic cardiac contracture reflected as enhanced LVEDP and increases in ET-1 release and CPP as compared to glucose group (*P* < 0.05). Also postischemic cardiac 15-F2t-isoprostane, a specific index of ROS-induced oxidative stress, was higher in low glucose group that was associated with higher cardiac lipid transporter CD36 protein expression than that in glucose group. Propofol significantly reduced IS and improved cardiac function and reduced cardiac ET-1 and CPP as well as CD36 in glucose group but not in low glucose group, indicating that enhancement of ROS induced oxidative stress by low glucose plays a key role in compromising propofol cardioprotection. Further, both hemodynamic and biochemical findings signaled a remarkably attenuated myocardial injury and enhanced cardiac function recovery by TMZ treatment in the LG+P+TMZ group as compared to the LG group and the LG+P group. Together, these results for the first time demonstrated that low glucose concentration as seen during tight glycaemic control may not only promote postischemic myocardial ROS formation through enhancing FA oxidation and exacerbating myocardial IRI but also play a critical role in unmasking propofol cardioprotection against IRI.

15-F2t-IsoP, one of the most abundant F2t-isoprostanes, was firstly reported by Morrow and colleagues in 1990 [33, 34], which is produced* in vivo* and has been intensively used as a sensitive and reliable marker of oxidative stress [33, 34]. Thus, measurement of 15-F2t-IsoP concentration should potentially allow for exploration of the role of ROS in the pathophysiology of myocardial ischemic diseases. Our previous study has demonstrated that 15-F2t-IsoP per se can exacerbate myocardial IRI evidenced as elevated CK-MB release, increased IS, and concomitant cardiac dysfunction [31]. In the same study, 15-F2t-IsoP, applied before or present in high concentration during ischemia, can lead to an increase in effluent ET-1 concentration during reperfusion in the isolated rat heart [31]. ET-1 has been known as one of the most potent vasoconstrictors and postulated to be attributable to the progression of myocardial IRI [35]. We also have found that 15-F2t-IsoP generation during myocardial ischemia reperfusion in an isolated rat heart model can be inhibited by treatment with 2,6-diisopropylphenol (propofol), which led to improved postischemic cardiac functional recovery [29]. Another study done by our group has also determined that antioxidant therapy with Salvia miltiorrhiza during cardiac surgery significantly reduces the lipid peroxidation product malondialdehyde levels in the plasma with concomitant reductions in ET-1 concentrations and improved postoperative cardiac function in patients [36]. Despite these findings of 15-F2t-IsoP in myocardial diseases, no evidence exists to show propofol treatment effects on the alterations of both 15-F2t-IsoP and ET-1 concentrations during myocardial IRI under the context of different concentrations of glucose. The current study extended previous study and observed a significant reduction in ET-1 (Figure 3(a)) and 15-F2t-IsoP (Figure 4(a)) concentration in propofol treatment groups under glucose condition but not under low glucose condition. It is plausible that antioxidant therapy with propofol may have reduced ET-1 release during myocardial ischemia reperfusion through attenuating ROS induced oxidative stress reflected as reduced 15-F2t-IsoP production under normal glucose condition. However, under low glucose condition, the potentially increased FA metabolism subsequent to increased CD36 expression may result in increased oxidative stress which compromised propofol cardioprotection through its antioxidant property. During reperfusion, the significant increase in cardiac ET-1 release occurred early at 1 min after reperfusion (Figure 3(a)), which proceeded the changes in CPP which did not significantly increase at Re-1 min (data not shown) until after 10 min of reperfusion (Figure 3(b)). This suggests that postischemic ET-1 release is, at least in part, responsible for the increase in CPP.

The significant increases of 15-F2t-IsoP in all experimental groups at early reperfusion (1 min) as compared to that at baseline indicate increased oxidative stress during early reperfusion. However, at the end of reperfusion (60 min), 15-F2t-IsoP concentration decreased to the same level as that at baseline which was consistent with our previous study which showed that 15-F2t-IsoP level was continuously increased during ischemia and early reperfusion but decayed quickly after about 30 min during reperfusion in patients undergoing coronary artery bypass grafting [37]. In contrast, ET-1 levels remained elevated at least 24 hours after cardiopulmonary bypass in patients [36]. The postischemic myocardial IS was assessed 60 min after reperfusion with concomitant measures of LV diastolic performance [38]. It should be noted that although Langendorff method is an ideal experimental model utilized to assess cardiac function and metabolism* ex vivo*, the measurement of right ventricular function which contributed to overall cardiac function is not assessed that causes some limitations in this study.

In this study, we detected that isolated rat hearts perfused with low glucose were more vulnerable to myocardial injury. Low glucose exhibited a significant myocardial injury evidenced by dramatically increased IS, CK-MB, and cardiac dysfunction compared to glucose group and this was tightly associated with increased 15-F2t-IsoP production and higher cardiac CD36 protein expression. CD36 is a transmembrane sarcolemmal protein considered as a major mediator of FA transport and participated in enhancing cardiomyocyte FA uptake and subsequently regulating FA oxidation rates in the hearts. There is a growing body of evidence finding that CD36 overexpression can facilitate FA oxidation whereas CD36 ablation can lead to reduced FA uptake and FA oxidation rates which is compensated by increased glucose oxidation and consequently prevent IRI [39]. Our data found that ROS production, reflected as 15-F2t-IsoP generation, was significantly increased in rat heart with low glucose and associated with increased CD36 expression indicating that low glucose concentration at 5.5 mM may result in increased FA oxidation and subsequently lead to lipid peroxidation production and render the heart susceptible to myocardial IRI. The fact that cardiac contractile dysfunction reflected as increased LVEDP and decreased LVDP was accompanied by increased CD36 level is in line with the viewpoint of Ussher and Lopaschuk [40] which suggests that accelerated FA oxidation can strongly affect perioperative cardiac function recovery. The translocation of CD36 can be regulated by both glucose metabolism and cardiac contraction and the dramatic reduction of CD36 after perfusion in propofol and TMZ treated group may be due to rapid redistribution from the functional lipid raft to nonfunctional nonraft membrane as suggested [41], but further in depth mechanistic study in this regard is merited.

Propofol has chemical structural similarity to that of the phenol-based free radical scavenger vitamin E and possessed antioxidant property [42]. Propofol at the concentrations 2–4 *μ*g/mL failed to achieve cardioprotective effect on coronary surgery patients [43]. However, propofol, when infused in high doses, may potentially impair the metabolic functions of blood vessels and muscles of various organs and subsequently lead to apparent heart failure, renal failure, and metabolic acidosis, which is known as propofol infusion syndrome [44]. In our study, propofol applied at 5 *μ*g/mL displayed a reduction of contracture development evidenced by increased LVDP function while decreased LVEDP confirmed that propofol can improve postischemic myocardial functional recovery. Our finding is in line with findings by others who showed that propofol attenuated contracture development through significantly facilitating energy metabolic recovery which may provide tolerance to lethal cardiac IRI [45].

Another important finding of this study is the correlation between different glucose concentrations and propofol cardioprotection against IRI. To our knowledge, this is the first study to compare the cardioprotective effect of propofol on isolated hearts perfused with low glucose (5.5 mM) and normal glucose (8 mM) concentration. Propofol under glucose 8 mM showed a robust myocardial protection effect. Whereas its cardioprotective effects vanished, the hearts were perfused at low glucose at 5.5 mM. Propofol had no significant effect on ameliorating myocardial injury between the LG and LG+P groups indicating that the abolishment of cardioprotective effect with propofol may be a result of low glucose induced enhancement of ROS production due to the increase of FA oxidation which exceeded propofol's antioxidant capacity. Our finding differs from that of Wang et al. [41] as they also showed that hearts exposed to propofol can increase glucose oxidation and markedly decrease FA oxidation. This discrepancy may be partially due to the fact that FA oxidation increased by low glucose surpasses the decrease by propofol. However, the exact mechanism is unclear and deserves further study.

Our finding that low glucose concentration-mediated cancellation of propofol cardioprotection can be restored by the addition of the partial FA oxidation inhibitor TMZ with concomitant reduction of postischemic oxidative stress provides indirect evidence that low concentration glucose may induce increased FA oxidation and exacerbate oxidative stress during myocardial IRI. Further, we showed that the protective effect of TMZ on postischemic myocardial damage and function recovery was more evident in low glucose group than in normal glucose group.

Propofol has been studied extensively for its cardioprotective effect [46]. However, that propofol treatment against IRI by modulating FA oxidation using TMZ has not been reported. The contribution of TMZ in propofol-mediated cardiomyocytes protection is addressed for the first time by using Langendorff perfused model. TMZ, a partial FA oxidation inhibitor, is well established to have efficient effect against ischemic damage as a result of increased glucose oxidation through selectively suppressing key enzyme of FA oxidation [47] and be regarded as an effective adjunctive treatment against global ischemia reperfusion [48]. The present study showed that TMZ supplementation restored isolated heart with low glucose concentration sensitivity to propofol cardioprotection by decreases in myocardial IS and 15-F2t-IsoP generation and enhancement in cardiac function which is aligned with studies from Lopaschuk et al. [49] and Fragasso et al. [50] as they also found that TMZ can improve functional recovery and diminish injury to ischemic myocardium. The novel finding that suppressing FA oxidation by TMZ facilitated propofol cardioprotection under low glucose may provide a potential direction on clinical therapies to combat global myocardial ischemia. The findings of the current study combined with our previous work on the cardioprotection effect of propofol suggest that a combined strategy with propofol and FA oxidation antagonism designed to specifically inhibit FA oxidation could pave the way for a therapeutic approach for amelioration of cardiac injury.

The current experiment data presented herein supporting the hypothesis that low glucose concentration induced the formation of myocardial ROS through enhancing FA oxidation may represent the major mechanism by which propofol lost its cardioprotective effect against IRI. This may provide a mechanism whereby the effectiveness of propofol cardioprotection in patients is not consistent. Our study suggests a novel therapy against myocardial IRI under low glucose that single therapy could not achieve satisfied cardioprotection but the combination use of propofol and TMZ can get better recovery. The future work will have to be performed to fully characterize the relationship of low glucose concentration and propofol cardioprotection in the pathogenesis of IRI and TMZ applied as anti-ischemic agent requires further evaluation in the laboratory and clinical setting.

## 5. Conclusions

In summary, isolated rat hearts with low glucose concentration at 5.5 mM lost sensitivity to propofol treatment through enhancing FA oxidation and oxidative stress and TMZ supplementation restored the sensitivity of isolated rat hearts to propofol that was concomitant with diminished myocardial IS, CK-MB release, ET-1, and 15-F2t-IsoP concentration as well as improved cardiac function.
